# Alteration of Lipid Bilayer Electrical Potential by Phytochemicals and Synthetic Analogs: Implications for Cellular Function

**DOI:** 10.3390/biom16030342

**Published:** 2026-02-24

**Authors:** Svetlana S. Efimova, Quan Minh Pham, Huong Thi Thu Trinh, Long Quoc Pham, Olga S. Ostroumova

**Affiliations:** 1 Institute of Cytology of Russian Academy of Science, Tikhoretsky Ave. 4, St. Petersburg 194064, Russia; efimova@incras.ru; 2 Institute of Chemistry, Vietnamese Academic of Science and Technology (ICH, VAST), 18 Hoang Quoc Viet, Hanoi 113000, Vietnam; minhquanaries@gmail.com (Q.M.P.);; 3 Laboratory of Biophysics, Institute for Advanced Study in Technology, Ton Duc Thang University, 19 Nguyen Huu Tho Street, Ho Chi Minh City 700000, Vietnam; phamquoclong@tdtu.edu.vn; 4 Faculty of Pharmacy, Ton Duc Thang University, 19 Nguyen Huu Tho Street, Ho Chi Minh City 700000, Vietnam

**Keywords:** phytochemicals, bioactive compounds, polyphenols, flavonoids, alkaloids, saponins, lipid membranes, membrane boundarypotential, membrane dipole potential, lipid packing stress

## Abstract

Phytochemicals, including flavonoids, stilbenoids, alkaloids, terpenoids, and structurally related synthetic small molecules, exhibit a broad spectrum of beneficial pharmacological effects. These effects stem not only from interactions with specific protein targets but also from their capacity to modify the physical properties of biological membranes. A key membrane property influenced by these plant-derived compounds is the electrical potential drop at the membrane–water interface, which plays a crucial role in numerous cellular processes. Changes in membrane potential impact the function of embedded proteins and ion channels, thereby modulating cell signaling, transport, and pharmacological responses. This review compiles data on how diverse plant and synthetic small molecules alter membrane physical characteristics, particularly the dipole component of the boundary potential in lipid bilayers primarily composed of phosphatidylcholine, a predominant membrane lipid in mammals and fungi. In-depth analysis of structure–activity relationships in this context elucidates how various structural modifications affect the compounds’ ability to shift membrane electrical potential. Understanding these relationships can pinpoint molecular features that drive membrane interactions and facilitate the discovery and design of more potent dipole-modifying agents with therapeutic potential.

## 1. Introduction

### 1.1. Origin of Membrane Dipole Potential

Cell membranes act as semipermeable barriers, separating the intracellular space from external surroundings while maintaining cellular integrity and compartmentalization. They facilitate cellular processes, regulate interactions, and sustain the transmembrane electrochemical potential crucial for signal transduction. The selective transport of ions creates a charge imbalance, generating an electrical potential difference (Δφ) across the membrane, typically ranging from 10 to 100 mV [1]. This potential influences membrane proteins like voltage-gated Na^+^, K^+^, and Ca^++^ channels [2], and can be measured experimentally with electrodes [3,4].

Another type of electrostatic potential associated with membranes is the boundary potential, which consists of two primary components: the surface potential (φ_s_) and the dipole potential (φ_d_). Biological membranes typically contain 10–20% negatively charged lipids. For example, mammalian cell membranes include phosphatidylserine [5,6,7], while bacterial membranes are primarily made up of phosphatidylglycerol and cardiolipin [8,9,10,11]. Mitochondrial membranes and those of late endosomal compartments are also characterized by a significant concentration of negatively charged lipids [12,13]. The magnitude of φ_s_, typically ranging in the tens of millivolts, is influenced by both the negative charges of membrane lipids and the degree to which these charges are neutralized by surrounding ions in the solution [1,14]. Due to the presence of negatively charged lipids in the membrane, φ_s_ facilitates the accumulation of cations near the membrane surface while reducing the concentration of anions [15]. Although measuring the exact value of φ_s_ is challenging, it can be approximated using the Gouy–Chapman–Stern model [16,17,18] or by determining the potential at the sliding plane, known as the electrokinetic potential (ζ) [18,19,20,21]. Numerous studies have addressed the impact of φ_s_ in ion transport processes, as outlined in a comprehensive review by Ermakov [22].

Regardless of membrane surface charge, the bilayer’s hydrophobic region has more positive potential compared to the surrounding solution, and this potential jump at the membrane boundary is called a dipole, φ_d_ [14,23,24,25,26,27]. It arises from the orientation of inherent lipid dipoles—particularly those from carbonyl groups and headgroups—along with the adjacent layer of water molecules at the lipid–water interface [28]. φ_d_ significantly increases permeability of phospholipid membranes to anions over cations, affecting protein conformation and function. Liberman and Topaly first proposed φ_d_ at the membrane/solution interface [29], while Chladky and Haydon provided experimental evidence in 1973 [30]. Later, Ketterer et al. [31] developed a model that incorporates the interaction potential energy of a hypothetical ion with both dipoles and membrane charges. Benz and co-authors introduced a generalized electrostatic potential profile that includes both φ_s_ and φ_d_ [32]. This approach remains relevant today.

### 1.2. Methods for Assessing the Membrane Dipole Potential

**Figure 1:**
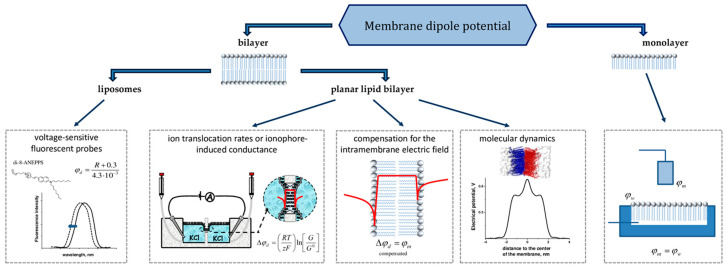
Schematic representation of the common existing methodological approaches to evaluate changes in the membrane dipole potential.

The magnitude of φ_d_ depends on the chemical structure of the lipids constituting the membrane [26,33,34,35,36,37]. Although the absolute value of the potential at the membrane/solution interface cannot be directly measured, it can be estimated using variety of methods and models (Figure 1).

The simplest method for quantitatively assessing the φ_d_ is the lipid monolayer technique [27,38,39]. The change in potential difference is measured after spreading a monolayer of lipid onto the surface of a Langmuir trough. It is crucial to note that the potential difference obtained through the monolayer technique for a given lipid composition significantly exceeds the values determined using methods involving planar lipid bilayers or lipid vesicles. Several factors may account for this discrepancy. These include the possibility that the φ_d_ values for the monolayer are overestimated, that the φ_d_ values for the bilayer are underestimated, or that the monolayer may not serve as a sufficiently accurate model to represent half of a bilayer [40]. The φ_d_ values of these two structures should not be directly compared. Therefore, in this review, all analyzed values were sourced from studies that conducted measurements using the bilayer techniques.

In terms of monitoring changes in the φ_d_, a simple and reliable method is through the use of voltage-sensitive fluorescent probes incorporated into lipid vesicles [29,40,41,42,43,44,45,46]. A common assumption in estimating φ_d_ with fluorescent potential-sensitive dyes is that the dye itself does not influence the φ_d_ value. Moreover, this technique relies on calibration with other methodologies. Despite this, the method offers an undeniable advantage regarding its applicability to measure φ_d_ in cell membranes [47,48,49,50].

Planar lipid bilayers serve as the most practical model system for experimentally evaluating changes in φ_d_ caused by ions and small molecules. The most commonly used approaches with lipid bilayers include determination of Δφ_d_ from ion translocation rates [40], ionophore-induced conductance [40,51], the compensation for the intramembrane electric field [52,53], and molecular dynamics calculations [54,55,56,57,58,59,60]. A method used to determine the ratio between the conductances of a hydrophobic anion (tetraphenylborate) and cation (tetraphenylphosphonium or tetraphenylarsonium) is complex and not free from artifacts [61]. The method—based on the measurement of ionophore-induced conductance of lipid bilayers, initially applied by Andersen et al. [51] to investigate changes in φ_d_ influenced by the plant flavonoid phloretin—was later refined in subsequent research [62]. However, the conductance-based approach has limitations, including the assumption that its value remains unaffected by factors unrelated to the electrical potential distribution. For instance, this method overlooks the impact of membrane fluidity on the mobility of charged complexes, which could influence the results. The compensation method is particularly useful for analyzing the asymmetry in potential distribution resulting from the adsorption of charged particles or dipole-modifying substances that cannot permeate through the membrane [52].

Additionally, the literature data supports the use of dielectric spectroscopy, cryo-electron microscopy and atomic force microscopy as alternative approaches to estimate φ_d_ [63,64,65,66]. It is noteworthy that the φ_d_ value can vary considerably depending on the estimation technique employed.

## 2. Membrane Dipole Potential Significance

The φ_d_ is vital for cellular function, progressively decreasing along the secretory/endocytic pathway: plasma membrane >> lysosome > Golgi > endoplasmic reticulum. Mitochondrial membranes maintain slightly higher φ_d_ levels than lysosomes [67]. This suggests φ_d_ plays a key role in membrane recognition, assisting protein sorting and docking based on dipole moments. During the cell cycle, φ_d_ peaks in the G1 phase due to increased cholesterol, then declines by 8–10% in the S and G2/M phases as lipids reorganize for cell division. These shifts contribute to early-cycle hyperpolarization and mitotic depolarization, ensuring ion flux and protein interactions necessary for cell cycle progression. Cholesterol adjustments largely drive these changes, linking φ_d_ regulation to crucial proliferation checkpoints [50]. Studies also show oxidative stress in human erythrocytes significantly elevates φ_d_ levels [48]. Interestingly, natural antioxidants like flavonoids may reduce φ_d_. Metabolic disorders, such as Smith–Lemli–Opitz syndrome, disrupt sterol biosynthesis and cause cholesterol precursor buildup, severely impacting φ_d_. This highlights its importance in understanding disease mechanisms [68].

φ_d_ regulates ion transport rates across biological membranes, influencing the gating of hERG K^+^ channels, OmpF porin, and KcsA channels [69,70,71,72,73]. Its impact on the selectivity of KvAP and GluR channels, connexin 32 hemichannel, SecYEG bacterial channel, ion channel produced by Vpu protein of HIV-1, and CNGA2 channel can stem from interactions between polar groups, dipole motifs, and membrane electrostatic potential [74,75,76,77,78,79,80]. This also applies to model systems like gramicidin A, alamethicin, syringomycin E, surfactin, amphotericin B, cecropin A, polymyxin B, and nisin [81,82,83,84,85,86,87,88,89,90,91,92,93,94,95,96].

φ_d_ heavily impacts the function of various membrane proteins, such as receptor tyrosine kinases of ErbB family, ion pumps like Na^+^-K^+^-ATPase and Ca^++^-ATPase, phospholipases A2 and C, the serotonin 1A receptor, and the binding of the pre-sequence of cytochrome c oxidase subunit IV (p25) to membranes [41,49,59,97,98,99,100,101,102,103,104,105]. The inhibition of Na^+^-K^+^-ATPase by ether lipids [106] may result from higher φ_d_ in ether lipid membranes compared to ester ones [28]. Additionally, *Clostridium perfringens* α-toxin aggregation on membranes is linked to an increase in φ_d_ [107].

Localized variations in φ_d_, particularly in cholesterol- and sphingolipid-rich membrane microdomains, may influence the function of specific peptides and proteins [55,108,109,110,111], including amyloid-β-peptides linked to Alzheimer’s disease [112]. Changes in membrane proteins due to lipid–protein interactions can be analyzed using φ_d_ as a sensitive marker. Additionally, φ_d_ measurements are useful for evaluating membrane protein purification, providing insights into dipolar environment shifts, protein function, and solubilization efficiency [113].

Extensive evidence highlights the role of φ_d_ in processes linked to viral activity. Elevated φ_d_ levels enhance bilayer stability by repelling cations necessary for hemifusion stalk formation, while modifiers reduce φ_d_ to facilitate processes such as viral entry or exocytosis [41]. The simian immunodeficiency virus fusion peptide interacts with membranes to influence φ_d_, which in turn impacts the degree of membrane fusion induced by the peptide [114]. Cholesterol has been shown to strengthen the affinity of the HIV GP41 fusion peptide for model membranes, accelerating lipid mixing rates through its effects on φ_d_ [115]. Adjustments to φ_d_ have been reported to directly influence the binding affinity of HIV fusion peptides to membranes [59]. Similarly, the antiviral effectiveness of 25-hydroxycholesterol against HIV may be tied to alterations in lipid membrane properties, including changes in φ_d_ [116]. φ_d_ serves as a key modulator in interactions between the HIV protease inhibitor saquinavir and membranes [117]. Gp41-derived peptides, such as sifuvirtide, enfuvirtide, and T-1249, which inhibit HIV-1 fusion, show a reduction in φ_d_ for erythrocyte and lymphocyte membranes [118,119]. Additionally, a cholesterol-conjugated derivative of C34, an HIV-1 fusion inhibitor peptide, significantly decreases φ_d_ while enhancing the peptide’s antiviral potency via membrane interactions [120]. Peptides from the S2 domain of SARS-CoV (770–788 and 1185–1202), associated with the fusion peptide and pre-transmembrane domain, respectively, can also decrease φ_d_ [121]. These effects are strongly dependent on membrane lipid composition, and combinations of such peptides may create synergistic impacts. This implies that alterations in φ_d_ influence SARS-CoV’s fusion process. For SARS-CoV-2, ether phospholipids have been found to alter the conformation of its fusion peptide and influence its binding to hybrid membranes, indicating that φ_d_ may significantly impact its fusion mechanism as well [122]. The binding affinity of the hemagglutinin fusion peptide has been observed to increase with higher φ_d_ levels [59].

The interaction of drugs and biologically active substances with membranes depends on φ_d_, and drugs by themselves also affect φ_d_ value [117,123]. φ_d_ regulates cell-penetrating peptide insertion into membranes [124] and inversely correlates with penetratin concentration in the cytoplasm [125,126]. Atorvastatin, an HMG-CoA reductase and cholesterol synthesis inhibitor, lowers φ_d_ [125]. Increased φ_d_ enhances bacitracin permeation [127], while antibiotic nisin significantly alters φ_d_ in negatively charged membranes [128]. Changes in φ_d_ may explain the antibacterial effects of polyalanine peptides [129]. Insecticide lindane, general and local anesthetics, and dexibuprofen-loaded nanoparticles also modulate φ_d_ [56,92,130,131,132,133,134,135,136,137]. Pregnanolone’s membrane partitioning is governed by φ_d_ [123]. Certain isoniazid derivatives reduce φ_d_ in membranes resembling *Mycobacterium tuberculosis*’s cell wall [138]. Natamycin binding and aggregation decrease φ_d_ [139]. Ru(III) complexes’ anticancer activity is linked to their lipid-specific dipole-modifying properties in normal and cancer cell membranes [140]. Procyanidin B3, picolinamide, verapamil, and arbutin lower φ_d_ [141,142,143,144], while φ_d_ changes impact piroxicam’s kinetics [145]. The antifungal activity of cationic gemini surfactants may stem from their dipole-modifying effect [146,147].

Literature findings indicate that φ_d_ can either inhibit or enhance the membrane insertion of amphiphilic antimicrobial peptides. This effect is contingent upon the orientation of the peptide’s hydrophobic dipole moment, positioning φ_d_ as a critical determinant of their antimicrobial efficacy alongside the surface charge of cellular membranes [59,148]. The dual regulation of surface charge and φ_d_ offers promising avenues for addressing microbial resistance mechanisms, particularly those linked to reductions in the negative surface charge. This can be achieved through the targeted modulation of the dipole component using small molecules.

## 3. Small Molecules to Disturb Membrane Dipole Potential

The investigation of the role of φ_d_ in diverse biological processes, alongside the quest for pharmacologically active compounds capable of enhancing the efficacy of various membranotropic drugs, has become an increasingly prominent area of focus within biology and pharmacology. Consequently, the continuous development and diversification of molecular tools—specifically, small molecules designed to facilitate precise and predictable modifications of the φ_d_—remains a critical objective in advancing this field.

In general, φ_d_ is related to the dipole moment of lipids, their packing density in the bilayer, and the membrane dielectric constant, as described by the Helmholtz equation for a planar capacitor [23,24,36]. It is believed that the incorporation of small molecules affects the dipole moment of lipids by virtue of the fact that they possess their own dipole moments in lipid bilayers, by alteration of membrane hydration, as well as by changing the surface density of dipoles [14,51,149,150,151,152,153]. A strong dependence of the φ_d_ on lipid packing stress and vice versa should also be taken into account [154,155,156].

Understanding how small molecules influence lipid membranes and their link to pharmacological activity is crucial for creating new drugs, particularly natural compounds targeting cell membranes directly to minimize drug resistance.

This review summarizes the available information about the modulation in the φ_d_ of lipid bilayers composed of phosphatidylcholines caused by small molecules of a different chemical nature. Phosphatidylcholines were chosen as an abundant membrane lipid species in eukaryotic cells [157,158].

### 3.1. Polyphenolic Compounds

Over 8000 different phenolic compounds have been identified, with new discoveries adding to this number each year [159,160]. Flavonoids and stilbenes are often studied in the context of drug discovery [161,162,163]. Findings from both preclinical and clinical studies reinforce the protective role of polyphenols in aging cells [164,165], cardiovascular [166], neurodegenerative [167], and oncological diseases [168]. This beneficial effect is largely attributed to their potent antioxidant properties, which stem from the presence of numerous hydroxyl groups. The broad and detailed classification of flavonoids highlights their remarkable structural and functional diversity. This underscores the importance of investigating the functional characteristics of flavonoids and identifying the key structural components that drive specific biological activities.

**Figure 2:**
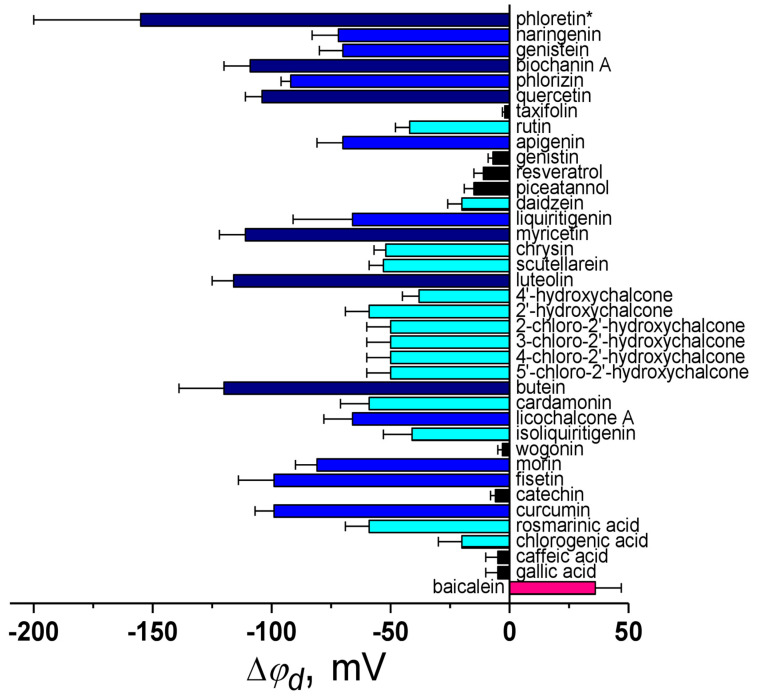
The action of various polyphenols on the dipole potential of phosphocholine membranes is explored. The mean maximum changes in the dipole potential (Δφ_d_) are taken from [14,51,93,149,150,153,169,170,171]. * The estimated Δφ_d_ value in the presence of phloretin is derived by consolidating and averaging data from [14,51,149,150]. The deviation from the mean Δφ_d_ value observed in experiments is presented as an error bar, and it is taken from the corresponding reference for a given compound or represents the maximum difference between mean Δφ_d_ values from different cited studies. The column color depends on the strength of the dipole-modifying ability of the compound: pink—slight increase in φ_d_ (20 ≤ Δφ_d_ < 60 mV), black—negligible effect on φ_d_ (|Δφ_d_| < 20 mV), cyan—slight decrease in φ_d_ (−20 ≥ Δφ_d_ > −60 mV), blue—moderate dipole-modifying efficiency (−60 ≥ Δφ_d_ > −100 mV), and dark blue—significant decrease in φ_d_ (Δφ_d_ ≤ −100 mV).

Figure 2 presents a summary of literature findings regarding the dipole-modifying capacity of polyphenols. It illustrates how the compounds influence φ_d_ of membranes composed of phosphatidylcholines. A correlation analysis using octanol/water partition coefficients (log*D*) and dipole moment values (μ), as presented in the relevant cited sources, shows no correlation between |Δφ_d_| and log*D* for polyphenols (Spearman’s rank correlation coefficient is equal to 0.1). At the same time, a significant correlation exists between |Δφ_d_| and dipole moment (μ) values (correlation coefficient is equal to 0.5).

**Figure 3:**
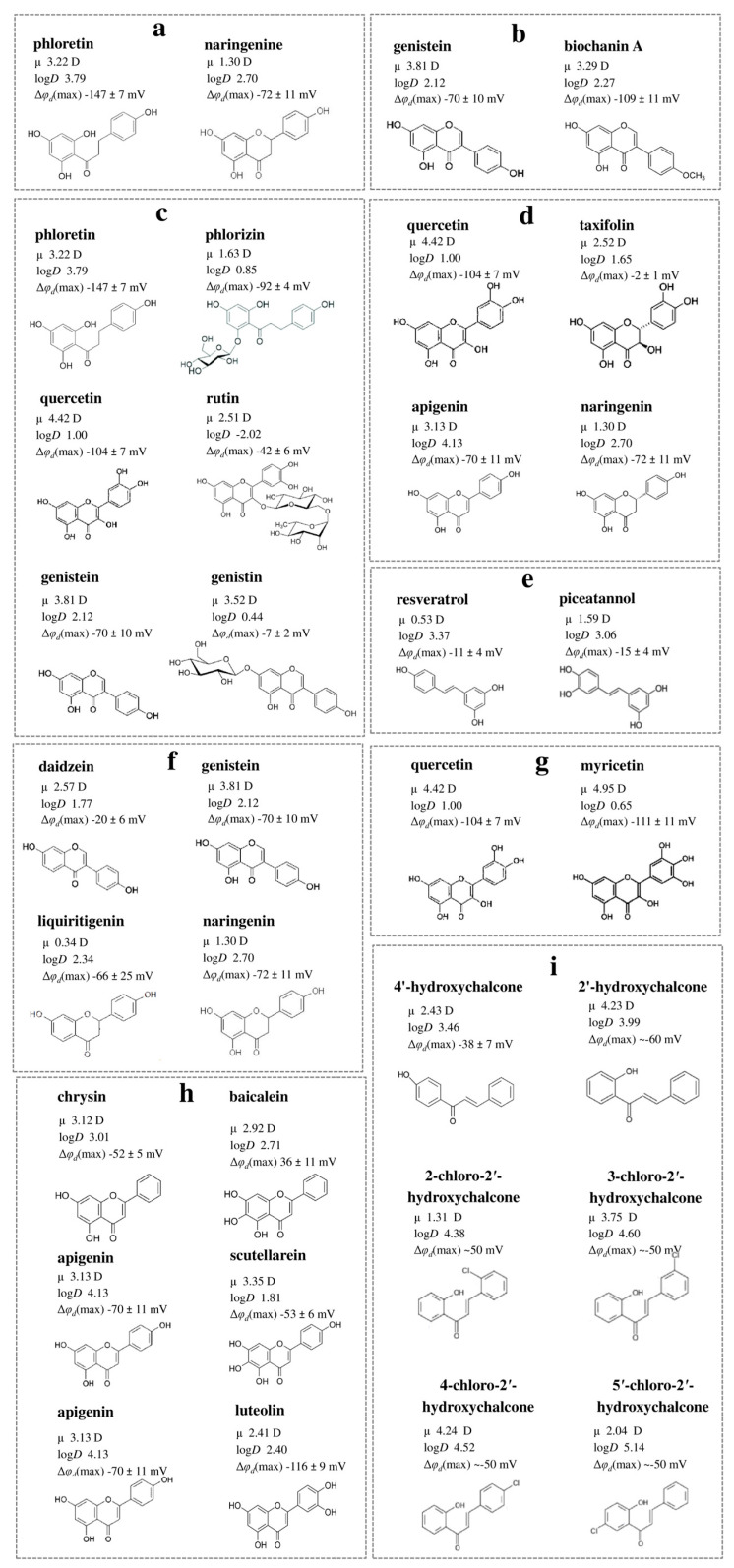
The relationship between the chemical structure of polyphenols and their dipole-modifying ability: (**a**) phloretin and naringenin; (**b**) genistein and biochanin A; (**c**) phloretin and phlorizin, quercetin and rutin, genistein and genistin; (**d**) quercetin and taxifolin, apigenin and naringenin; (**e**) resveratrol and piceatannol; (**f**) daidzein and genistein, liquiritigenin and naringenin; (**g**) quercetin and myricetin; (**h**) chrysin and baicalein, apigenin and scutellarein, apegenin and luteolin; (**i**) 4′-hydroxychalcone and 2′-hydroxychalcone, 2-chloro-2′-hydroxychalcone and 3-chloro-2′-hydroxychalcone, 4-chloro-2′-hydroxychalcone and 5′-chloro-2′-hydroxychalcone. The log*D*, μ, and Δφ_d_ values are presented according to [93,149,153,169,170,173]. Abbreviations: log*D*—the value of the logarithm of the octanol/water partition coefficient at pH 7.4; *μ*—molecular dipole moment; Δφ*_d_*(max)—the maximum changes in the dipole potential of neutral membranes enriched with phosphatidylcholine (detailed information about the fatty acid composition of membrane-forming lipid and the method of Δφ*_d_* estimation can be found in the relevant references [93,149,153,169,170].

By analyzing the chemical structures of polyphenols, especially those closely related as structural analogs, alongside the magnitude of changes that they induce in the φ_d_, the following patterns were observed:

(1) The closing of a heterocycle in the naringenin molecule compared to phloretin leads to a decrease in the molecule’s dipole-modifying ability by 75 mV due to a reduction in both the magnitude of μ (by about 2 D) and log*D* (by more than 1) (Figure 3a). Therefore, the key factors responsible for the observed effects are the molecule’s polarity and hydrophobicity.

(2) Methylation of the hydroxyl group at the 4′-position of the B-ring in biochanin A compared to genistein leads to a significant increase in the dipole-modifying ability, although μ and log*D* values of the isoflavones are close (Figure 3b). More probably, this modification affects the molecule’s orientation, causing the dipole moment of biochanin A to align more parallel to the normal than to the membrane surface. The assumption that not only the magnitude but also the orientation of μ in the membrane plays a key role aligns well with the existing data [172].

(3) Glycosylation at the 6′-position of the A-ring, 3-position of the C-ring and 7-position of the A-ring of phloretin, quercetin, and genistein, respectively, results in a significant decrease in log*D* value (by 1.7–3) and consequently in dipole-modifying ability (by about 60 mV). In the case of chalcones and flavonols, the effect may also be attributed to differences in polarity between the glycosylated and nonglycosylated forms (by about 1.5–2 D) (Figure 3c).

(4) The hydrogenation of a double bond in the heterocycle of flavonol quercetin to obtain flavanonol taxifolin leads to a significant reduction in μ (by about 2 D) and loss of dipole-modifying ability, indicating that molecular polarity is a key factor (Figure 3d). In contrast, the same modification with flavone apigenin to get flavanone naringenin, compared to the other, also causes a similar decrease in μ but does not affect the Δφ_d_ value (Figure 3d). This difference may be attributed to the influence of the presence/absence of the hydroxyl group in the 3-position on the orientation of the flavonoid molecules within the lipid bilayer.

(5) Stilbenes, such as resveratrol and piceatannol, whose polyphenol rings are connected by a hydrocarbon linker lacking heteroatoms, generally do not exhibit the ability to affect the φ*_d_* independently of the number of hydroxyl groups. This is likely due to their relatively low μ, suggesting that the polarity of polyphenol molecules is markedly determined by the degree of oxidation of the linker between the rings (Figure 3e).

(6) An additional hydroxyl group at the 5-position of the A-ring in the isoflavone genistein compared to daidzein leads to an increase in μ (by 1.7 D) and |Δφ_d_| value (by 50 mV). In contrast, the same modification in the flavanones liquiritigenin and naringenin resulting in an increased μ (by about 1 D) does not enhance the dipole-modifying ability. These findings highlight the crucial role of molecular orientation in the lipid bilayer affected by the double bond in the heterocycle or the position of the B-ring (Figure 3f).

(7) The additional hydroxyl group at the 5′-position of the B-ring in myricetin compared to quercetin slightly increases μ but decreases log*D*, which results in similar dipole-modifying ability of the flavonols (Figure 3g).

(8) Flavones chrysin and baicalein, which are similar in structure, lipophilicity, and μ, differ by a hydroxyl group at the 6-position of the A-ring and exhibit opposite effects in terms of the φ*_d_* value: baicalein increases φ*_d_*, while chrysin decreases it (Figure 3h). Baicalein and scutellarein, which differ in one hydroxyl group at the 4′-position of the B-ring, also demonstrate opposite effects in terms of φ*_d_* (Figure 3h). These observations suggest that the key factor influencing the dipole-modifying ability of flavones is the orientation of their dipole moments within the membrane. A similar modification of apigenin to obtain scutellarein results in a significant decrease in log*D*, but not dipole-modifying ability, which also indicates the dependence of the orientation of the molecule in the bilayer on the number and localization of hydroxyl groups in the flavone structure (Figure 3h). Hydroxylation at the 5′-position of the B-ring of apigenin to get luteolin leads to a decrease in μ (by about 1 D) and log*D* value (by 1.7) but an increase in dipole-modifying activity, which also indicates the predominance of orientation of flavones in the membrane over their polarity and hydrophobicity (Figure 3h).

(9) A hydroxyl group at the 2′-position of the A-ring of chalcone is more preferential than at the 4′-position, while the localization of the chlorine atom does not matter (Figure 3i).

The biological activities of flavonoids and stilbenes are closely associated with their specific interactions with cell membranes. These compounds act as prominent modulators of various ion transport systems, including KCNQ channels [174], hERG channel [175], Na^+^-K^+^-2Cl^−^ cotransporter 1 [176], different subtypes of the voltage-dependent (K_V_) K^+^-channels [177], Ca^++^-activated K^+^ channels [178,179], ATP-sensitive K^+^ channels [180], voltage-gated Na^+^ channels [181], cystic fibrosis transmembrane conductance regulator [182], α_1_β_1_γ_2s_ GABA_A_ and ρ_1_ GABA_C_ receptors [183] and others. Through these modulatory functions, flavonoids and stilbenes exhibit significant therapeutic potential in treating conditions such as cardiovascular diseases, cancer, and cystic fibrosis. Additionally, the deep localization of polyphenols within membranes is known to greatly influence their effectiveness in combating diseases like cancer and neurodegenerative disorders [184,185].

Phloretin potentiates a lot of membranotropic antimicrobial agents by reducing φ_d_ [81,83,89,91,93,95,96]. The ability of butein, naringenin, cardamonin, 4′-hydroxychalcone, licochalcone A, and liquiritigenin to decrease the conductance of single ion channels produced by the antifungal cyclic lipopeptide syringomycin E was in agreement with their effects on φ_d_ of the lipid bilayers [93]. Chlorination of hydroxychalcone B-ring potentiates the ability of compound to diminish φ_d_ and improves its antimicrobial, antibiofilm, and antiproliferative activities, especially against aggressive breast cancer cell lines, leading to the consideration that the disturbance of the φ_d_ might have a main role in the implementation of biological action [169]. Protective effect of polyphenols from field horsetail extract on erythrocytes is believed to be attributed to the reduction of φ_d_ of cell membranes [186]. Anti-atherosclerotic activity of naringenin and naringin dihydrochalcone on blood cells might be related to their ability to reduce φ_d_ [187].

### 3.2. Thyroid Hormones and Xanthene Dyes

Thyroid hormones and xanthene dyes, though fundamentally distinct in their nature and functions, share a remarkable structural similarity: both consist of hydroxylated and halogenated benzene rings connected via an oxygen atom. Thyroid hormones, such as thyroxine and triiodothyronine, are iodinated derivatives of thyronine, whose core structure features two benzene rings bridged by an oxygen atom. These hormones primarily differ in the number of halogen substituents attached to their base structure. In contrast, xanthene dyes are synthetic compounds characterized by a three-ring system, where two benzene rings are joined by an oxygen-containing heterocyclic ring. Their structure also includes a benzoic acid group that forms a lactone ring with the xanthene core. Among these dyes, fluorescein stands out as a key representative; it undergoes halogenation to produce various derivatives. Xanthene dyes are widely used in medical applications due to their chromatic properties. Fluorescein, for example, plays a critical role in ophthalmology, where it is applied topically to assess corneal damage [188]. Similarly, Rose Bengal is utilized in the evaluation of liver function, showcasing the diverse clinical applications of these compounds [189]. The inclusion of halogen atoms significantly impacts the electron density within the benzene rings, thereby modifying the molecules’ dipole moments.

**Figure 4:**
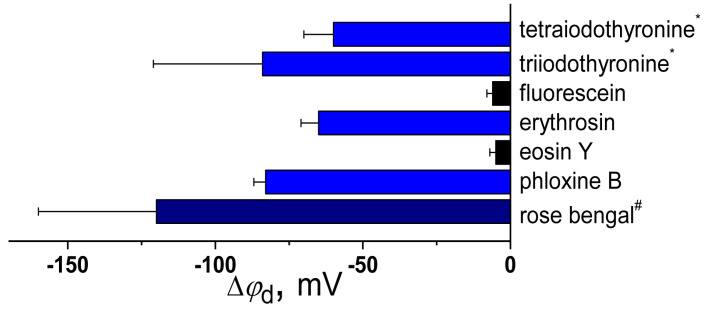
The effects of thyroid hormones and xanthene dyes on the dipole potential of phosphocholine membranes. The mean maximum changes in the dipole potential (Δφ*_d_*) are taken from [94,190]. The estimated Δφ_d_ values are derived by consolidating and averaging data from various cited sources, such as those provided for * tetraiodothyronine and triiodothyronine [94,190] and ^#^ Rose Bengal [94,190]. The deviation from the mean Δφ_d_ value observed in experiments is presented as an error bar, and it is taken from the corresponding reference for a given compound or represents the maximum difference between mean Δφ_d_ values from different cited studies. The column color depends on the strength of the dipole-modifying ability of the compound: black—negligible effect on φ_d_ (|Δφ_d_| < 20 mV), blue—moderate dipole-modifying efficiency (−60 ≥ Δφ_d_ > −100 mV), and dark blue—significant decrease in φ_d_ (Δφ_d_ ≤ −100 mV).

Figure 4 presents a summary of literature findings regarding the dipole-modifying capacity of thyroid hormones and xanthene dyes. There is a strong correlation between |Δφ_d_| and μ, and |Δφ_d_| and log*D* values for xanthene dyes (Spearman’s rank correlation coefficient is equal to 0.8 and 0.5 respectively). Similar to polyphenols, this might be associated with embedment of xanthene dyes dipoles into membrane.

**Figure 5:**
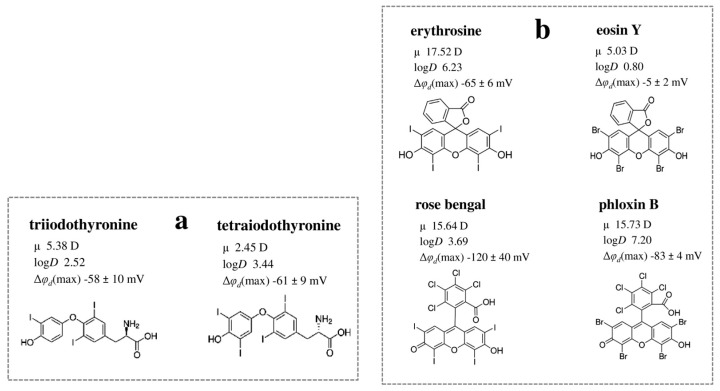
The relationship between the structure of thyroid hormones (**a**) and xanthene dyes (**b**) and their dipole-modifying ability. The logD, μ, and Δφd values are presented according to [94,173].

Tetraiodothyronine, which contains an additional iodine atom compared to triiodothyronine, exhibits a lower μ but higher lipophilicity, factors that likely account for the small differences observed in the dipole-modifying activity of these molecules (Figure 5a).

The dipole-modifying effect of fluorescein derivatives is determined by the polarity of their molecules and depends on the type of halogen substituents introduced into the fluorescein structure. Iodinated derivatives, such as erythrosine and Rose Bengal, exhibit a stronger ability to reduce φ*_d_* compared to brominated derivatives like eosin Y and phloxin B, respectively (Figure 5b). The presence of a tetrachlorobenzene moiety significantly enhances dipole-modifying ability; thus, phloxin B is capable of reducing φ*_d_*, whereas eosin Y lacks this effect, and Rose Bengal has a more pronounced effect than erythrosine (Figure 5b).

The integrin αvβ3 on the plasma membrane and the truncated nuclear receptor TRα1 (p28) in the inner mitochondrial membrane act as thyroid hormone receptors, initiating their non-genomic effects [191]. Studies show triiodothyronine increases inward rectifier K^+^-channel activity by increasing their open probability, likely contributing to shorter action potential duration in hyperthyroidism [192]. Additionally, thyroid hormones’ activation of the Na^+^/H^+^ exchanger and Ca^++^-ATPase may explain their neuroprotective effects [193]. It is hypothesized that thyroid hormones induce a general disturbance in transmembrane dipolar organization, which could represent a fundamental aspect of their non-genomic mechanisms [194].

Upon photoirradiation, xanthene dyes, particularly Rose Bengal, phloxine B, erythrosine B, and eosin Y, have been demonstrated to induce K^+^ leakage from *Staphylococcus aureus* cells and cause dissipation of the membrane potential. These effects are primarily attributed to the production of reactive oxygen species [195]. Interestingly, while both thyroid hormones and xanthene dyes lead to a reduction in φ_d_, they influence syringomycin E ion channels through distinct mechanisms. Thyroid hormones, such as thyroxine and triiodothyronine, exert their effects primarily by altering membrane elasticity, whereas Rose Bengal, phloxine B, and erythrosine influence the ion channels by affecting the bilayer electrostatics [94].

### 3.3. Saponins and Sapogenins

Saponins’ structural diversity underpins their physico-chemical, biological, and pharmacological properties, making them valuable in food, cosmetics, and pharmaceuticals [196]. They possess various effects, including anti-inflammatory, antipyretic, anti-nociceptive, anticancer, and anti-allergic activities [197]. Their hemolytic activity arises from interactions with cholesterol in erythrocyte membranes [198]. Saponins also play a key role as adjuvants in vaccine production [199,200,201]. Research highlights certain types—ginsenosides and diosgenins—as potential chemopreventive agents, and saikosaponins, glycyrrhizins, cycloartanes, dammaranes, oleananes, spirostanes, and furostanes for their anticancer potential [202,203,204,205,206].

**Figure 6:**
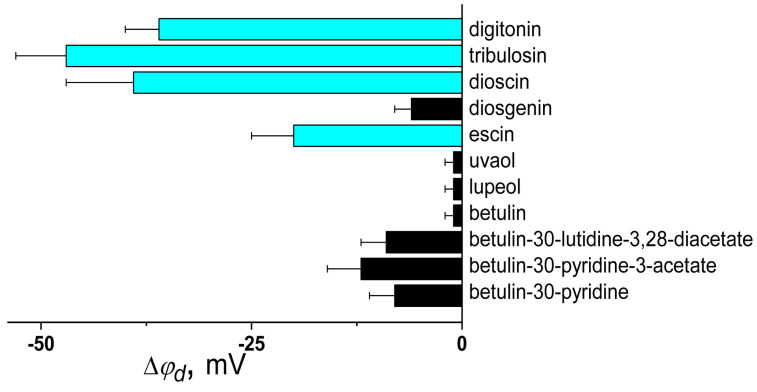
The effect of saponins and related compounds on the dipole potential of phosphocholine membranes. The mean maximum changes in the dipole potential (Δφ*_d_*) are taken from [152,207]. The deviation from the mean Δφ_d_ value observed in experiments is presented as an error bar, and it is taken from the corresponding reference for a given compound. The column color depends on the strength of the dipole-modifying ability of the compound: black—negligible effect on φ_d_ (|Δφ_d_| < 20 mV), cyan—slight decrease in φ_d_ (−20 ≥ Δφ_d_ > −60 mV).

Figure 6 summarizes literature findings on the dipole-modifying properties of saponins and related compounds. It illustrates the impact of these compounds on the φ_d_ of phosphatidylcholine bilayers and includes a correlation map linking their potential-modifying ability to the μ or log*D* values. Only glycosylated saponins, such as digitonin, tribulosin, dioscin, and escin, exhibit a moderate ability to influence φ_d_. In contrast, all sapogenins, including diosgenin, uvaol, lupeol, and betulin, regardless of whether their core structure is steroidal or triterpenoid, do not affect φ_d_ (Figure 6). This indicates the significance of saponin’s ability to interact with water molecules absorbed onto the membrane. This fact is consistent with an absence of correlation between |Δφ*_d_|* and μ-values and a significant correlation between Δφ*_d_* and log*D* of saponins (Spearman’s rank correlation coefficient is equal to 0.1 and −0.7 respectively).

**Figure 7:**
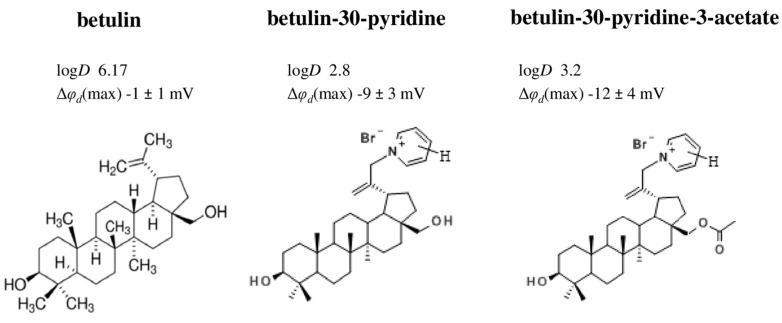
The relationship between the structure of betulin derivatives and their dipole-modifying ability. The log*D*, μ, and Δφ_d_ values are presented according to [152,207].

Also, this shows that pyridine derivatives of betulin have more pronounced effects on φ*_d_* value compared to betulin (Figure 7). Taking into account a decrease in log*D* (by about 3) and the high dipole moment of the pyridine moiety (2.26 D), we can hypothesize that the observed increase in dipole-modifying ability is related to an increase in molecular polarity.

Saponins have the ability to physically extract cholesterol from cell membranes, particularly from lipid rafts, which play a vital role in functioning of various ion channels and cell signaling. The depletion of cholesterol caused by saponins can indirectly influence other crucial membrane lipids, such as phosphatidylinositol 4,5-bisphosphate (PIP_2_) [208], for example, resulting in the inhibition of BK_Ca_ channels [209]. Ginsenoside-Rd, a glycosylated saponin, appears to prevent the progression of atherosclerosis, likely through the inhibition of voltage-independent Ca^++^ channels [210].

### 3.4. Alkaloids and Related Antibacterial Compounds

Plant alkaloids have been found to exhibit various effects, including vasodilatory, antihypertensive, antiarrhythmic, anesthetic, and analgesic properties [211,212]. Additionally, some alkaloids demonstrate antiproliferative, antibacterial, and antioxidant activities [213]. Given the amphiphilic nature of most of alkaloids, it is hypothesized that one potential mechanism of their action involves interactions with lipid cell membranes.

**Figure 8:**
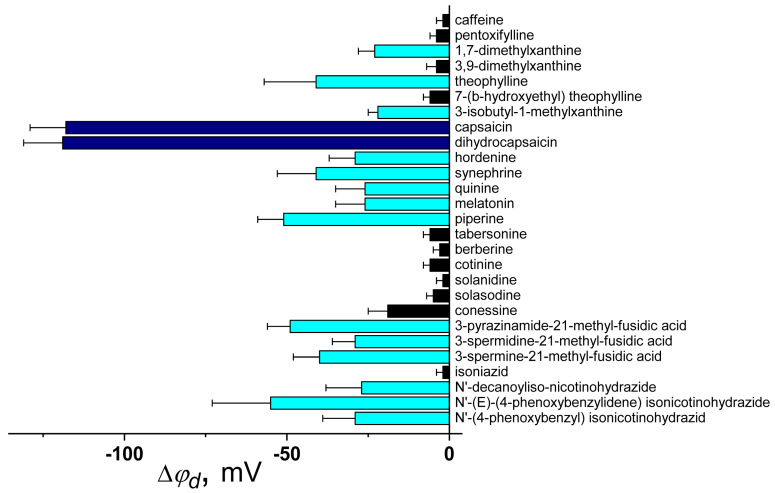
The influence of alkaloids and related compounds on the dipole potential of phosphocholine membranes is explored. The mean maximum changes in the dipole potential (Δφ*_d_*) are taken from [138,151,214]. The deviation from the mean Δφ_d_ value observed in experiments is presented as an error bar; it is taken from the corresponding reference for a given compound. The column color depends on the strength of the dipole-modifying ability of the compound: black—negligible effect on φ_d_ (|Δφ_d_| < 20 mV), cyan—slight decrease in φ_d_ (−20 ≥ Δφ_d_ > −60 mV), and dark blue*—*significant decrease in φ_d_ (Δφ_d_ ≤ −100 mV).

Figure 8 summarizes available literature findings on the dipole-modifying ability of various alkaloids and nitrogen-containing antibiotics.

By examining the chemical structures of alkaloids and related compounds, particularly those that are structurally analogous, alongside the magnitude of changes they induce in φ_d_, certain patterns have been identified.

**Figure 9:**
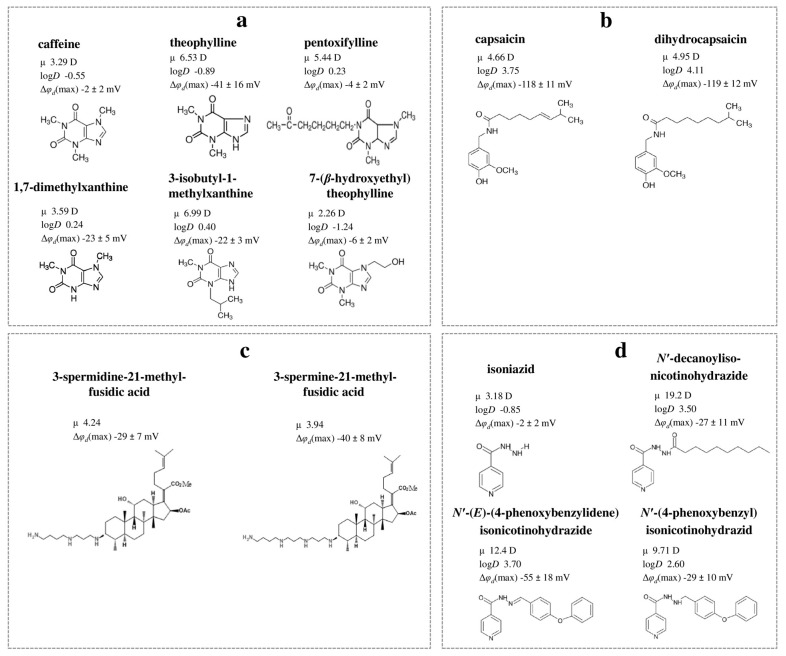
The relationship between the structure of alkaloids and related compounds and their dipole-modifying ability: (**a**) xanthines; (**b**) capsaicin and dihydrocapsaicin; (**c**) spermin derivatives; (**d**) isoniazid derivatives. The log*D*, μ, and Δφ_d_ values are presented according to [138,151,214].

(1) A moderate correlation exists between |Δφ_d_| and μ for xanthines (Spearman’s rank correlation coefficient is equal to 0.4), likely due to these molecules integrating their dipoles into lipid bilayers, despite their relatively low log*D* values (about −1–0). The absence of a methyl group at the 3-position of 1,7-dimethylxanthine compared to caffeine does not affect the μ value although it is accompanied by an increase in dipole-modifying ability (by 20 mV) (Figure 9a). At the same time, the absence of the CH_3_-group at the 7-position of theophylline compared to caffeine leads to a significant increase in the μ-value (by more than 3 D) and the |Δφ_d_| value (by 40 mV) (Figure 9a). An addition of isobutyl moiety to 1,3-dimethylxanthine results in a slight increase in dipole moment compared to theophylline but a minor reduction in dipole-modifying ability (Figure 9a). Unlike 7-(β-hydroxyethyl)theophylline, which is not characterized by a larger μ than caffeine, the oxohexyl radical at the 1-position of pentoxifylline increases the polarity but not the dipole-modifying ability of the molecule compared to caffeine (Figure 9a). All these observations indicate that the orientation of the dipole moment of the xanthine molecule in the membrane is more important than its absolute value.

(2) The pronounced ability of benzylamine protoalkaloids capsaicin and dihydrocapsaicin to reduce the φ*_d_* is attributed to their high lipophilicity and polarity (Figure 9b). A hydrogenation of a double bond in the side chain of dihydrocapsaicin compared to capsaicin does not influence its dipole-modifying due to branching at the end of the “tail” of both molecules, which should have a disordering effect on the lipid bilayer and consequently decrease the dipole density.

(3) Fusidic acid derivatives with promising antibacterial activity, presented in Figure 9c, reduce the membrane boundary potential by approximately 30–40 mV, which is likely related to their relatively high μ values (about 4 D).

(4) The incorporation of decanoyl, phenoxybenzylidene, or phenoxybenzyl radical into the well-known antituberculosis antibiotic isoniazid structure results in the enlargement of both µ (by 7–16 D) and log*D* value (by about 4), thereby promoting the ability of molecules to penetrate membranes and exhibit their dipole-modifying properties (Figure 9d).

The literature indicates that alkaloids affect ion channels via lipid-mediated actions [215,216,217,218]. Capsaicin reduces membrane rigidity, influencing voltage-gated Na^+^ channels, ASIC1a, ASIC3, ENaC, P2x2, and GABA receptors [215,216,217,218]. Similar effects are noted on MscL, Kv2.1, and NaV channels [216]. Capsaicin impact Kv2.1 gating via alterations in the physicochemical properties of the membrane [219]. It also alters channels like CFTR, BK/Maxi-K, TRP, and L-type Ca^++^ channels by modifying the lipid matrix rather than directly interacting with proteins [216]. These findings emphasize alkaloids’ role in altering membrane elasticity and suggest they may regulate ion channels via transmembrane electrical potential changes, as seen with some antimicrobial agents [95,96,151].

### 3.5. Anesthetics

Local anesthetics have been utilized for several decades, yet their exact molecular mechanisms of action continue to be a subject of scientific inquiry. It is generally recognized that their analgesic effect is achieved by inhibiting voltage-gated Na^+^ channels in nerve fibers [220]. To accomplish this, local anesthetics must penetrate the plasma membrane and bind to specific sites located within the inner part of the channels [221,222,223]. Furthermore, the correlation between the lipid–water partition coefficients of the anesthetics and their ability to interact with membrane lipids indicates that membrane lipids themselves may serve as key targets, potentially enhancing the drugs’ clinical efficacy [224]. Although general anesthetics have been used in surgical practice for over 150 years, their precise molecular mechanism remains largely unclear. It is widely accepted that the anesthetic effect is primarily linked to the membrane-mediated action of these drugs on ion channels, such as GABA_A_ receptors and K2P channels [225].

**Figure 10:**
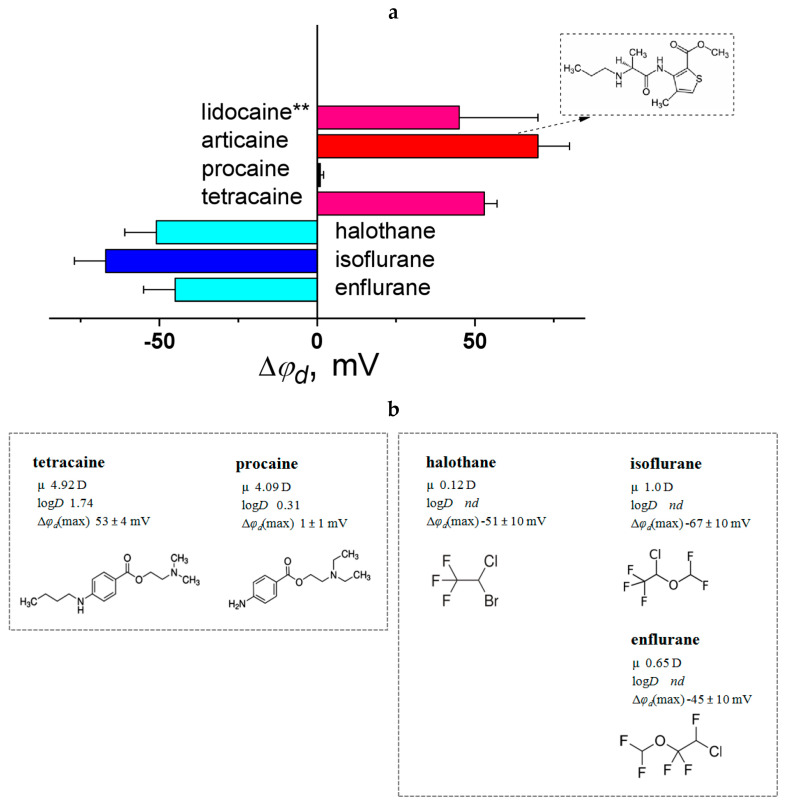
(**a**) The action of anesthetics on the dipole potential of phosphocholine membranes is explored. The mean maximum changes in the dipole potential (Δφ_d_) are taken from [56,92,135]. ** The estimated Δφ_d_ value in the presence of lidocaine is derived by consolidating and averaging data from various cited sources [56,92]. The deviation from the mean value of Δφ_d_ observed in the experiment is presented as an error bar; it is taken from the corresponding reference for certain compounds or represents the maximum difference between the mean values of Δφ_d_ obtained in different cited studies. The color of the column depends on the strength of the dipole-modifying ability of the compound: red*—*moderate enhancement in φ_d_ (60 ≤ Δφ_d_ < 100 mV), pink—slight increase in φ_d_ (20 ≤ Δφ_d_ < 60 mV), black—negligible effect on φ_d_ (|Δφ_d_| < 20 mV), cyan—slight decrease in φ_d_ (−20 ≥ Δφ_d_ > −60 mV), blue—moderate dipole-modifying efficiency (−60 ≥ Δφ_d_ > −100 mV). Inset—the chemical structure of articaine. (**b**) The relationship between the structure of general anesthetics and their dipole-modifying ability. The log*D*, μ, and Δφ_d_ values are presented according to [56,92,135] (*nd*—not determined).

Figure 10 presents a summary of literature findings regarding the dipole-modifying capacity of local and general anesthetics.

A correlation exists between the chemical structures of amino esters and their ability to modify dipole potential. Longer hydrocarbon side chains, as seen in tetracaine versus procaine (Figure 10b), enhance membrane dipole potential due to tetracaine’s greater hydrophobicity. This aligns with studies where molecular dynamics simulations show deeper tetracaine integration into the lipid bilayer compared to procaine [226]. However, molecular dynamics simulations examining lidocaine’s effects on φ_d_ diverge from electrophysiological data probably due to differences in experimental design. Simulations use short durations and insert molecules into the membrane, whereas in vitro studies add compounds to the surrounding solution. Consequently, bilayer concentrations are lower in vitro, and higher small molecule levels may increase their effects.

Tetracaine stands out as one of the uncommon dipole modifiers with the ability to markedly enhance membrane dipole potential. Combining tetracaine with dipole-reducing compounds provides an opportunity to investigate contrasting dipole-modifying effects, particularly in research focused on the regulation of pore-forming activities of antimicrobial agents. This highlights the significance of tetracaine as a valuable molecular tool.

Substitution of the bromine at the 2-position in halothane for the difluoromethoxy-group in isoflurane, or changing the localization of chlorine and fluorines between the 1- and 2-carbons in the ethane fragment (isoflurane versus enflurane) does not practically affect the dipole-modifying ability (Figure 10b).

Taking into account the diminishing in φ_d_ by general anesthetics might provide missing pieces of the puzzle in the membrane theory of general anesthesia [133]. 

### 3.6. Phosphodiesterase Type 5 Inhibitors

Benzenesulfamide derivatives, such as sildenafil and vardenafil, along with benzodioxolyl compounds like tadalafil, are well-known phosphodiesterase type 5 inhibitors used in treatment of erectile dysfunction [227]. These inhibitors primarily promote smooth muscle relaxation by facilitating the accumulation of cyclic guanosine monophosphate (cGMP). By blocking phosphodiesterase type 5 activity, cGMP levels rise, initiating a chain reaction through the activation of protein kinase G and lowering calcium concentrations. This process ultimately results in significant muscle relaxation [228].

**Figure 11:**
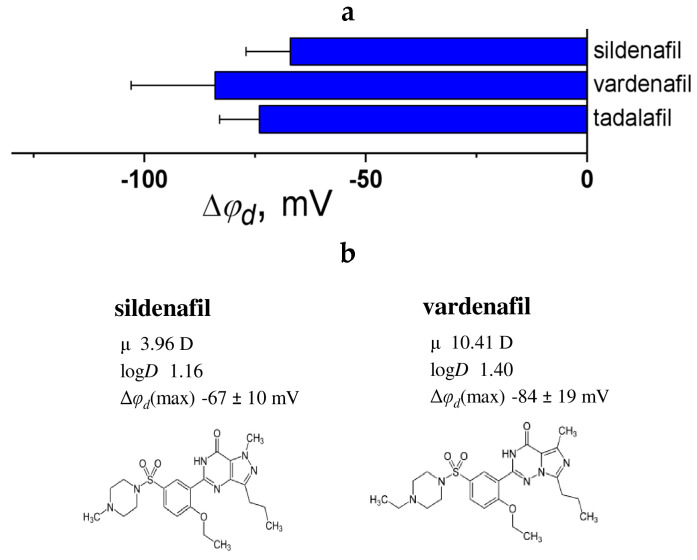
(**a**) The effect of phosphodiesterase type 5 inhibitors on the dipole potential of phosphocholine membranes is explored. The mean maximum changes in dipole potential (Δφ*_d_*) are taken from [229]. The deviation from the mean value of Δφ_d_ observed in the experiment is presented as an error bar; it is taken from [229]. The blue color of the columns corresponds to moderate dipole-diminishing efficiency of the compounds (−60 ≥ Δφ_d_ > −100 mV). (**b**) The relationship between the structure of compounds and their dipole-modifying ability. The log*D*, μ, and Δφ_d_ values are presented according to [229].

Figure 11a provides a comprehensive summary of findings from the literature concerning the ability of benzenesulfamide and benzodioxolyl derivatives to modify the dipole properties of phosphatidylcholine membranes.

Sildenafil and vardenafil reduce the φ_d_ by a comparable amount (70–80 mV), even though vardenafil has a considerably larger μ (by approximately 7 D) compared to sildenafil (Figure 11b). This suggests that the additional methyl group attached to the piperazine ring plays a significant role in influencing the molecule’s orientation within the lipid bilayer. To gain a clearer understanding of the relationship between the dipole-modifying effects of phosphodiesterase type 5 inhibitors and specific pharmacophore groups within these molecules, it is essential to investigate the membrane interactions of other compounds from this class of drugs.

The data collected permits the conclusion that, aside from the established mechanism of action characterized by smooth muscle relaxation through the accumulation of cyclic guanosine monophosphate (cGMP) [230], an alternative mechanism may be hypothesized for benzenesulfamide and benzodioxolyl derivatives. This alternative mechanism is proposed to involve their interaction with the lipid matrix of cellular membranes, enhancing the permeability for nitric oxide [231]. Increased nitric oxide diffusion subsequently stimulates guanylate cyclase activity, facilitating the conversion of guanosine triphosphate into cGMP [232].

### 3.7. Styryl Dyes

Styryl dyes, featuring a polymethine chromophore with conjugated double bonds, are rapid potential-sensitive membrane dyes [233]. Their optical response occurs within milliseconds, with fluorescence intensity and spectral profile influenced by transmembrane electric fields [234,235,236,237]. Styryl dyes are valuable for monitoring membrane-related processes in excitable cells [238]. However, while their spectral properties are well-documented, their interaction mechanisms with biological membranes remain unclear. A key limitation is the potential for these nanosensors to disrupt electrical charge distribution in the bilayer.

**Figure 12:**
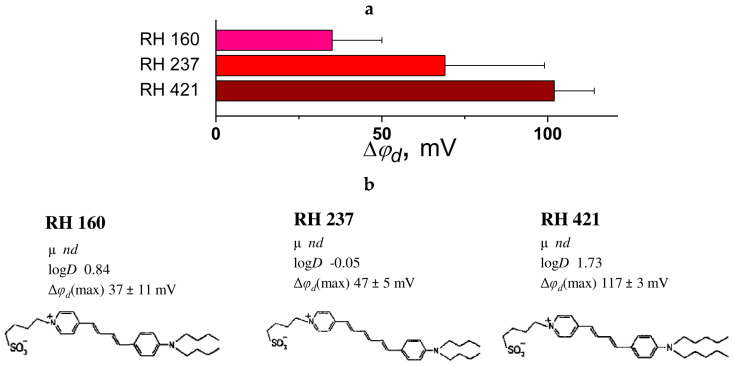
(**a**) The effects of styryl dyes on the dipole potential of phosphocholine membranes. The mean maximum changes in the dipole potential (Δφ_d_) are derived by consolidating and averaging data from [149,239]. The deviation from the mean Δφ_d_ value observed in experiments is presented as an error bar; it is taken from the corresponding reference for a given compound or represents the maximum difference between mean Δφ_d_ values from different cited studies. The color of the column depends on the strength of the dipole-modifying ability of the compound: burgundy—significant increase in φ_d_ (Δφ_d_ ≥ 100 mV), red—moderate enlargement in φ_d_ (60 ≤ Δφ_d_ < 100 mV), and pink—slight increase in φ_d_ (20 ≤ Δφ_d_ < 60 mV). (**b**) The relationship between the structure of styryl dyes and their dipole-modifying ability. The log*D*, μ, and Δφ_d_ values are presented according to [149] (*nd*—not determined).

Figure 12a presents a summary of literature findings regarding the dipole-modifying capacity of styryl dyes in phosphatidylcholine membranes.

The ability to enhance the φ_d_ diminishes in the order RH 421 > RH 237 ≈ RH 160. The dipole moment of these dyes is formed by a negatively charged sulpho-group located at the membrane–water interface and a positive charge, which is distributed in a chromophore [240]. It is interesting that the increase in the length of the linker between two aromatic rings in RH 237 compared to RH 160 does not produce noticeable changes in the |Δφ_d_| value likely due to the simultaneous increase in μ and decrease in log*D* (Figure 12b). An increase in the length of side chains in RH 421 compared to RH 160 significantly increases the dipole-modifying ability of the dye (by 80 mV), probably due to a higher log*D* value (by about 1) (Figure 12b).

The available literature emphasizes the need to reassess cell biology methodologies involving RH-series styryl dyes as potential-sensitive probes [241]. Due to the structural similarity between RH-series styryl dyes and ANEP, there arises a critical question regarding the feasibility of utilizing ANEP-based probes for such applications.

### 3.8. Chromonylallylmorpholine Derivatives

Research indicates that chromone-containing allylmorpholines exhibit strong selectivity for AChE and moderate antagonistic activity toward NMDA receptors [242], highlighting their potential as effective treatments for neurodegenerative conditions. Although the lipophilicity of these molecules is influenced by their functional groups, the morpholine ring itself contributes an optimal balance between lipophilic and hydrophilic properties, alongside drug-like characteristics favorable for therapeutic development [243].

**Figure 13:**
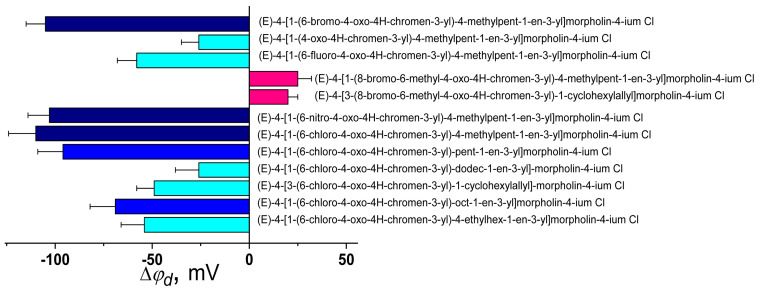
The influence of chromonylallylmorpholines on the dipole potential of phosphocholine membranes is explored. The mean maximum changes in the dipole potential (Δφ_d_) are taken from [244]. The deviation from the mean Δφ_d_ value observed in experiments is presented as an error bar. The color of the column depends on the strength of the dipole-modifying ability of the compound: pink—slight increase in φ_d_ (20 ≤ Δφ_d_ < 60 mV), cyan—slight decrease in φ_d_ (−20 ≥ Δφ_d_ > −60 mV), blue—moderate dipole-modifying efficiency (−60 ≥ Δφ_d_ > −100 mV), and dark blue—significant decrease in φ_d_ (Δφ_d_ ≤ −100 mV).

Figure 13 illustrates how chromonylallylmorpholines influence φ_d_ of membranes composed of phosphatidylcholines and also provide the correlation map between their potential-modifying ability and μ/log*D* values of modifier molecules.

**Figure 14:**
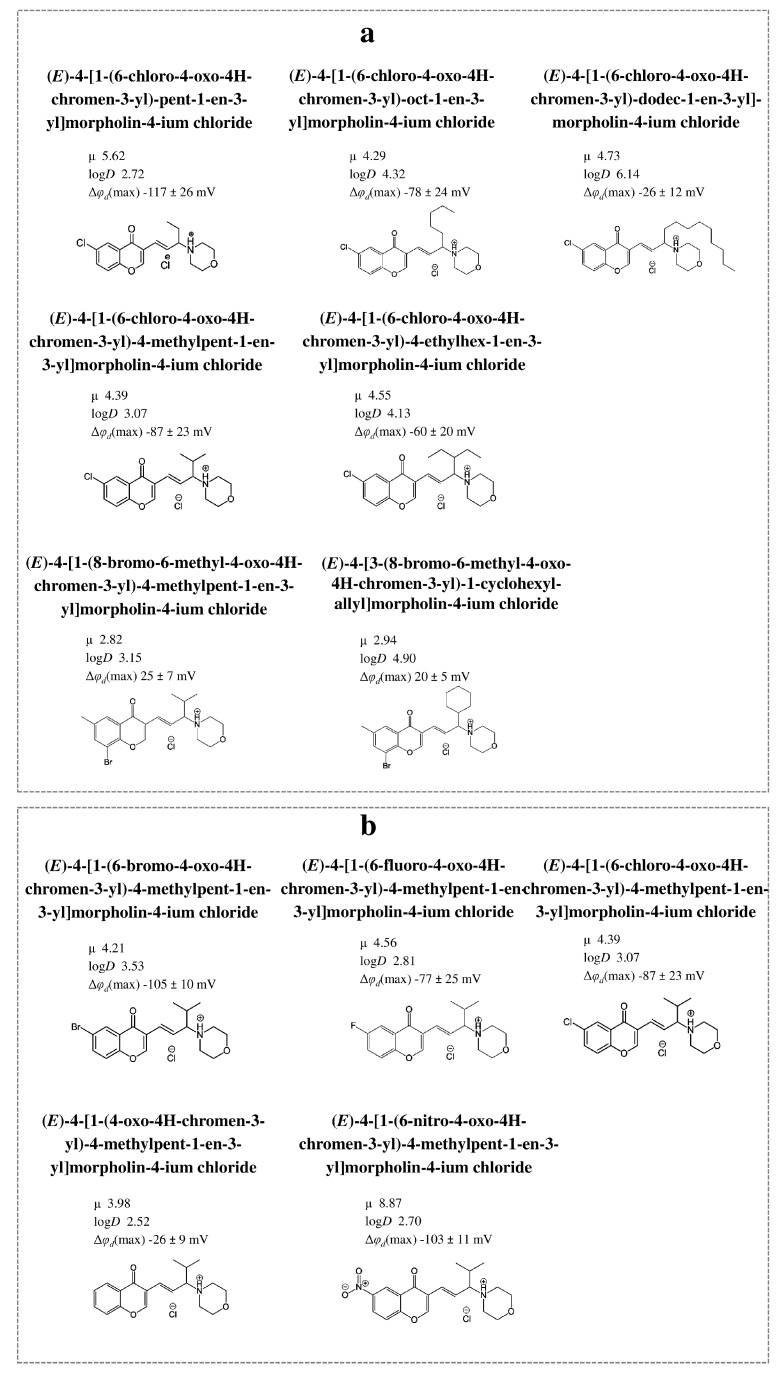
The relationship between the structure of chromonylallylmorpholines (**a**,**b**) and their dipole-modifying ability. The log*D*, μ, and Δφ_d_ values are presented according to [244].

There is a significant correlation between |Δφ_d_*|* and μ for chromonylallylmorpholines (Spearman’s rank correlation coefficient is about 0.5), which can be explained by the hypothesis that these molecules influence φ_d_ through their dipoles interacting with lipid bilayers, but at similar μ values, an increase in the length of the straight side chain (from 2 to 8 carbonyl atoms) or branched chain (from isopropyl to diethyl methyl) in the series of chromonylallylmorpholines is accompanied by an expected increase in log*D* and an unexpected decrease in dipole-modifying ability (by 70 and 60 mV, respectively) (Figure 14a). Moreover, there is a significant inverse correlation between Δφ_d_ and log*D* values of chromonylallylmorpholines (correlation coefficient is equal to −0.5). Probably, the elongation of the hydrophobic radical contributes to a decrease in the projection of μ onto the normal to the membrane surface.

The replacement of the halogen substituent at the 6-position for a hydrophobic methyl group and the introduction of a bromine atom at the 8-position also alter the orientation of the allylmorpholine molecule within the bilayer, and 8-bromo-6-methyl-derivatives acquire the ability to increase φ_d_, in contrast to their 6-bromo-analogs (Figure 14a). It is noteworthy that in this case the introduction of cyclohexane instead of isobutyl in the position does not affect the Δφ_d_-value despite the increase in log*D* (by about 2).

The type of halogen substituent (bromine, fluorine, and chlorine at the 6-position) does not significantly affect the impact of these compounds on the change in φ*_d_* (Figure 14b). The presence of an electron-withdrawing group at the 6-position of chromonylallylmorpholines is a key factor determining the molecule’s ability to reduce φ*_d_*. The unsubstituted derivative exhibits virtually no dipole-modifying activity compared to the halogenated analogs or the nitro-substituted compound (Figure 14b).

Acetylcholinesterase and NMDA receptors are membrane-associated proteins, and changes to the lipid matrix caused by chromonylallylmorpholines can influence their conformational transitions and functionality. The reduction in φ_d_ triggered by allylmorpholines leads to an increase in the conductance of gramicidin A channels [244].

### 3.9. 1,3-Thiazine, 1,2,3,4-Dithiadiazole, and Thiohydrazide Derivatives

Over the last decade, thiazines, thiadiazoles, and thiohydrazides have gained attention for their sedative, antimicrobial, antiviral, antifungal, and antitumor properties. Notably, phenothiazines like methylene blue and azure dyes show antimicrobial, neuroprotective, and anticancer effects by intercalating with DNA, inhibiting key enzymes, and modulating cellular redox states. They are also used in photodynamic therapy to generate reactive oxygen species targeting pathogens or tumors [245,246]. Similarly, 1,2,3-dithiazoles exhibit antifungal, antibacterial, antiviral, and anticancer activity through ring-opening to form disulfides that disrupt enzyme activity or induce apoptosis. They inhibit melanin synthesis, act as herbicidal agents via gibberellin oxidase inhibition, and show antifibrotic potential [247]. Thiohydrazides, precursors to thiadiazoles, demonstrate anti-inflammatory, antiparasitic, and antitubercular effects by inhibiting enzymes like InhA and EthR in mycobacteria [248]. Their 1,3,4-thiadiazole derivatives halt cancer cell growth and offer analgesic and anticonvulsant benefits. Additionally, thiadiazoles are being studied for Alzheimer’s disease, diabetes, and inflammation [245]. Emerging research highlights 1,2,3,4-dithiadiazoles as promising inhibitors of store-operated calcium channels [249,250]. Future clinical success relies on resolving issues such as their interaction with membrane lipids.

**Figure 15:**
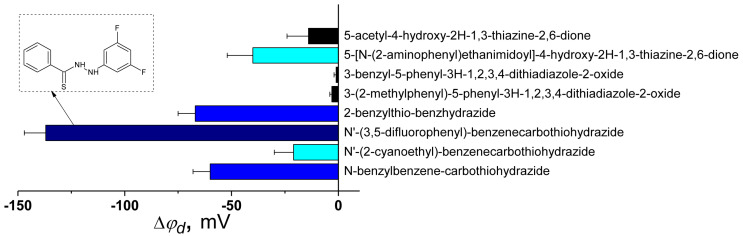
The effect of azoles on the dipole potential of phosphocholine membranes is explored. The mean maximum changes in the dipole potential (Δφ_d_) are taken from [251]. The deviation from the mean Δφ_d_ value observed in experiments is presented as an error bar. The color of the column depends on the strength of the dipole-modifying ability of the compound: black—negligible effect on φ_d_ (|Δφ_d_| < 20 mV), cyan—slight decrease in φ_d_ (−20 ≥ Δφ_d_ > −60 mV), blue—moderate dipole-modifying efficiency (−60 ≥ Δφ_d_ > −100 mV), and dark blue—significant decrease in φ_d_ (Δφ_d_ ≤ −100 mV). Inset—the chemical structure of *N*’-(3,5-difluorophenyl)-benzenecarbothiohydrazide.

Figure 15 presents a summary of literature findings regarding the dipole-modifying capacity of a series of thiazines, thiadiazoles, and thiohydrazides.

Based on the data obtained, it can be concluded that among the derivatives of thiohydrazides, containing not only nitrogen and sulfur atoms, but also fluorine, has the greatest ability to reduce the dipole potential of the membrane and has good prospects for use as a dipole-modifying agent. A decrease in the φ_d_ with *N*’-(3,5-difluorophenyl)-benzenecarbothiohydrazide adsorption facilitated the immersion of positively charged syringomycin E into the lipid bilayer and increases the pore-forming ability of the lipopeptide [251]. 

Literature indicates potential uses for derivatives of 1,2,3,4-dithiadiazole as novel calcium modulators, specifically inhibiting store-operated calcium entry [249,250,252], and the membrane-mediated component of such type of activity should be evaluated.

## 4. Summary

**Figure 16:**
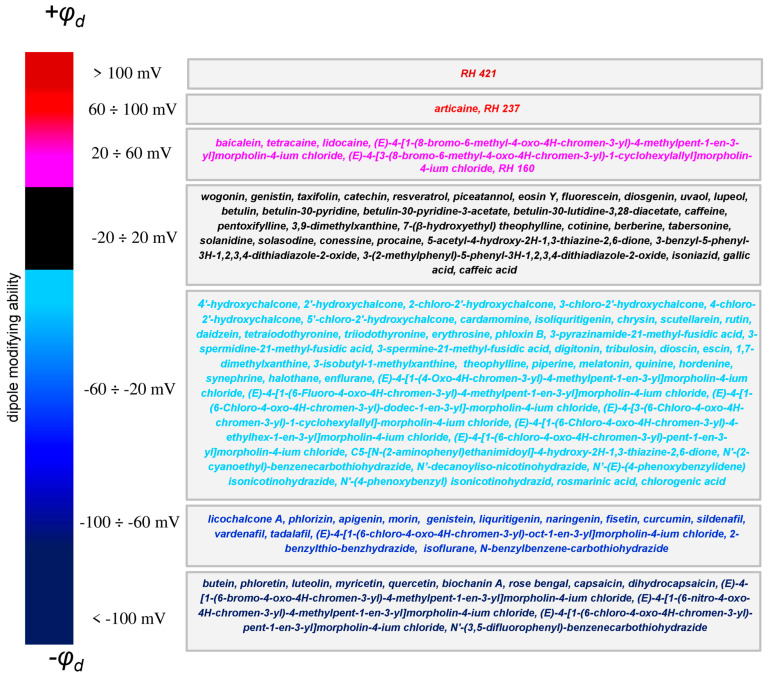
Classification of small molecules according to their dipole-modifying ability.

We categorized the compounds mentioned in this review into seven categories based on their dipole-modifying efficacy: agents that significantly increase φ_d_ (marked in burgundy); demonstrate moderate and slight enlargement in φ_d_ (marked in red and pink, respectively); do not practically affect φ_d_ (marked in black); slightly decrease φ_d_ (marked in cyan); have moderate dipole-diminishing efficiency (marked in blue); and significantly decrease φ_d_ (marked in dark blue) (Figure 16).

There are several compounds that are able to dramatically decrease membrane dipole potential (more than 100 mV): butein, phloretin, luteolin, myricetin, quercetin, biochanin A, Rose Bengal, capsaicin, dihydrocapsaicin, (*E*)-4-[1-(6-bromo-4-oxo-4H-chromen-3-yl)-4-methylpent-1-en-3-yl] morpholin-4-ium chloride, (*E*)-4-[1-(6-nitro-4-oxo-4H-chromen-3-yl)-4-methylpent-1-en-3-yl]morpholin-4-ium chloride, (*E*)-4-[1-(6-chloro-4-oxo-4H-chromen-3-yl)-4-methylpent-1-en-3-yl]morpholin-4-ium chloride, (*E*)-4-[1-(6-chloro-4-oxo-4H-chromen-3-yl)-pent-1-en-3-yl]morpholin-4-ium chloride, *N*’-(3,5-difluorophenyl)-benzenecarbothiohydrazide. Thus, chalcones, flavones, flavonols, isoflavones, halogenated xanthenes, benzylamines, and 6-halogen-substituted chromonylallylmorpholines are effective pharmacophores to reduce the dipole component of the membrane dipole potential. The decrease in the membrane dipole potential for these types of molecules is attributed to the incorporation of polar modifier molecules into the lipid bilayer. Structural modifications that affect not only the lipophilicity and polarity of compounds but also their orientation in the membrane should be taken into account. Molecular tools for significantly increasing the dipole potential are much more modest: this is only one stylyl dye, RH421.

The compiled repository of data on the effects of a wide range of low-molecular-weight natural and synthetic compounds on the physical properties of membranes of various compositions provides a valuable resource for gaining fundamental insights into lipid-mediated mechanisms of regulation of ion transport systems. It also enables the prediction of membrane activity of structurally related compounds and supports the development of combined antimicrobial agents comprising a pore-forming antibiotic and a small molecule potentiator.

**Figure 17:**
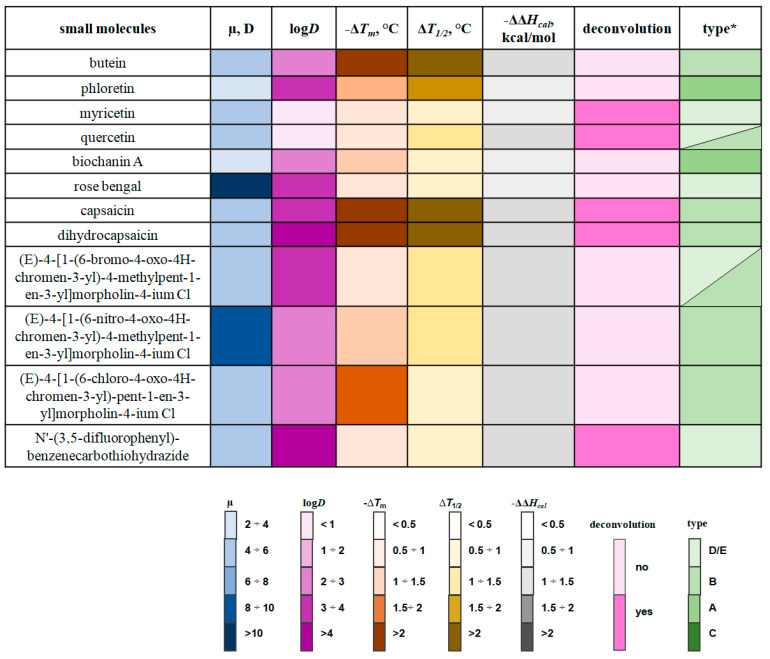
The molecular characteristics of the most effective dipole modifiers, their influence on various parameters of the dipalmitoylphosphocholine melting endotherm, and their potential localization within the membrane have been analyzed based on the classification system provided by Jain and Wu [256] (type*). The terms μ, log*D*, Δ*T_m_*, Δ*T*_1/2_, ∆∆*H_cal_* represent the molecular dipole moment, the logarithm of the octanol/water partition coefficient, changes in the main phase transition temperature, alterations in the half-width of the main peak, and variations in the enthalpy of dipalmitoylphosphocholine’s main phase transition. Data on the melting endotherm parameters of dipalmitoylphosphocholine in the presence of these dipole modifiers were compiled from studies [93,94,151,244,251,253,254,255]. Amphiphiles classified as types A, B, C, and D/E are proposed to localize in distinct regions of the membrane: specifically, near the C1–C9 atoms of hydrocarbon chains, the glycerol backbone, the C9–C16 portion of acyl chains in membrane lipids, and in proximity to the phosphocholine residues, respectively.

However, it is important to note that the incorporation of small molecules into the bilayer leads not only to changes in its electrical properties but also to alterations in lipid packing density. The influence of small molecules on lipid melting thermograms has been studied via differential scanning microcalorimetry of lipid vesicles [93,94,151,244,251,253,254,255]. Figure 17 highlights key molecular properties (μ and log*D* values) and parameters of dipalmitoylphosphocholine melting endotherms (changes in the main phase transition temperature (Δ*T_m_*), in the half-width of the main peak (Δ*T*_1/2_), in the enthalpy of the main phase transition (∆∆*H_cal_*), and the absence/presence of an additional shoulder on the main peak) when exposed to small molecules that significantly decrease membrane dipole potential (Δφ_d_ ≤ −100 mV). According to Jain and Wu [256], a certain pattern of alterations in the lipid melting thermogram can indicate the molecule’s membrane localization. In particular, a significant decrease in the *T_m_*, an increase in the *T*_1/2_, and a constant ∆*H_cal_* indicate localization of the guest molecule in the region of the C1–C9 atoms of the hydrocarbon chains of membrane-forming lipids (type A). If the ∆*H_cal_* also decreases and an additional shoulder appears on the main peak, the molecule is thought to be located in the glycerol region (type B). If the introduction of a modifier molecule is accompanied only by a decrease in the ∆*H_cal_*, then localization near the phosphocholine residue is assumed (type E or D, depending on the presence of a shoulder on the main peak). Small molecules that cause a dramatic decrease in melting temperature without significant changes in the *T*_1/2_ and the ∆*H_cal_* are believed to be located in the C9–C16 region of the acyl chains of membrane lipids. The right column in Figure 17 shows the most probable localization of modifier molecules in the lipid bilayer according to the classification of Jain and Wu [256]. Only in the presence of Rose Bengal do the parameters of dipalmitoylphosphocholine melting thermograms remain almost unchanged (Figure 17), which does not allow us to make assumptions about its significant immersion into the lipid bilayer. However, molecular dynamics simulations revealed deeper penetration of the dye into the membrane [257]. Thus, the most effective dipole modifiers are characterized by preferential localization at the membrane–water interface or in the region between the hydrophilic and hydrophobic parts of the lipid bilayer. Moreover, Figure 17 shows a possible relationship between the dipole-modifying and disordering abilities of butein, phloretin, quercetin, biochanin A, capsaicin, dihydrocapsaicin, (*E*)-4-[1-(6-nitro-4-oxo-4H-chromen-3-yl)-4-methylpent-1-en-3-yl]mor-pholin-4-ium chloride, (*E*)-4-[1-(6-chloro-4-oxo-4H-chromen-3-yl)-4-methylpent-1-en-3-yl]morpholin-4-ium chloride, and (*E*)-4-[1-(6-chloro-4-oxo-4H-chromen-3-yl)-pent-1-en-3-yl] morpho-lin-4-ium chloride, as indicated by the noticeable alteration in *T_m_* and *T*_1/2_.

## 5. Outlooks

Natural compounds from plants used in traditional medicine represent an important source for the search for membrane modifiers. Salvianolic acids, phytoconstituents from *Salvia miltiorrhiza* Bunge, having potential against coronary heart disease [258], might decrease φ_d,_ similar to rosmarinic acid. Among chalcone derivatives, boesenbergin A and 2′,4′-dihydroxy-6′-methoxy-3′,5′-dimethylchalcone, isolated from *Boesenbergia rotunda* and the buds of *Cleistocalyx operculatus* respectively [259,260], attract great attention. The high dipole-modifying activity of isoflavone tectorigenin, a main compound in *Belamcanda chinensis*, having strong analgesic and anti-inflammatory activities [261], flavone tectochrysin from *Pinus krempfii* Lecomte demonstrating acetylcholinesterase inhibitory activity [262], and analogs of curcumin, demethoxycurcumin, and bisdemethoxycurcumin, purified from *Curcuma longa*, which are dual inhibitors of protein tyrosine phosphatase 1B and α-glucosidase [263], may be proposed. Two flavonols, quercetin-3-*O*-methyl ether and kaempferol, for example, found in *Camellia chrysantha* [264] and various flavones, callistine A, eucalyptin, and 8-demethyleucalyptin, isolated from *Callistemon citrinus* [265], and flavones isolated from *Isodon ternifolius* [266], are also of great interest as membrane dipole modifiers. The dipole-modifying activity of luteolin tetramethyl ether, a chemical constituent of *Desmodium gangeticum* [267], and flavonols of *Orthosiphon stamineus* Benth., 5,7,3′,4′-tetramethylquercetin, 3′-hydroxy-3,5,7,4′-tetramethoxyflavone, and 3,5-dihydroxy-7,3′,4′-trimethoxyflavone [268], and isoflavanones from *Desmodium heterophyllum* (3*R*)–2′,4′,5,7-tetrahydroxy-6-methylisoflavanone and dalbergioidin [269], should be appropriately addressed. Paris saponin II from *Paris polyphylla* var. *chinensis* exhibiting cytotoxic effects against the MCF-7 human cancer cell line [270], and oleanolic triterpene saponins from *Panax bipinnatifidus* [271], ardinsuloside from *Ardisia insularis* [272], and saponin androseptoside A from *Polyscias guilfoylei* [273] might decrease φ_d_ similar to other glycosides of saponins. It was established that the alleviating atherosclerosis effect of kaempferol, widely distributed in a variety of Chinese herbal medicine, is related to inhibiting the Piezo1 channels and Ca^++^ influx by the flavonol [274]. Considering its high structural similarity with quercetin, myricetin and fisetin, which significantly reduce φ_d_, one can assume the significance of its dipole-modifying properties in the regulation of ion transport through biomembranes.

Literature data indicate that many novel dipole-modifying agents are expected to be discovered, with several compounds already shown to alter φ_d_ significantly. Thus, 3-phenylindole, an antimicrobial, greatly enhances K^+^-nonactin-induced membrane conductance, which might indicate a decrease in φ_d_ [275]. Carbonylcyanide phenylhydrazone in charged and uncharged forms alters boundary potential and φ_d_, inhibiting mitochondrial oxygen consumption and transport [276]. The calcium channel blocker verapamil changes φ_d_, affecting membrane translocation and ion partitioning [143]. Trehalose decreases φ_d_, while sucrose increases it, potentially by altering water molecules absorbed onto the lipid bilayer [277]. Arbutin diminishes φ_d_ as trehalose and phloretin but is believed to have an alternative mechanism of action [144]. *N*(alpha)-benzoyl-*L*-argininate ethyl ester chloride reduces φ_d_ more effectively than arginine because of its hydrophobic groups enhancing membrane insertion [278]. Procyanidins show biological activity linked to φ_d_ reduction, relevant for disease prevention and treatment [141]. Promising pharmacophores contained in essential oils ((*E*)-tagetone, (*Z)*-ocimenone, and thymol) and gallic acid may aid the discovery of new dipole-modifying agents [279,280].

A very important question is the possible relationship between changes in the elastic and electrical properties of the membrane [110,281,282]. The investigation into how small molecules can alter the elastic properties of membranes represents a distinct area of research that warrants further exploration. This line of inquiry could facilitate the identification of membrane modifiers useful for examining the regulation of mechanosensitive ion channels.

These findings suggest ongoing identification and characterization of new compounds with significant effects on φ_d_, with potential applications in medicine and biology.

## 6. Conclusions

Understanding the membrane role in transport processes—including the function of ion channels, pumps, carriers, and membrane–drug interactions—requires a thorough description of the electrostatic properties of the lipid bilayer. One of the key components of the membrane electrostatic landscape is the dipole potential, an internal component of electrical potential originated by the specific orientation of dipoles of phospholipid headgroups and membrane-associated water molecules. This often-overlooked potential resides within the hydrophobic core of the bilayer and plays a fundamental role in modulating membrane function by affecting protein conformation, membrane ion permeability, and, cell signaling and cycle. The measured value of the dipole potential is highly method dependent. However, from a practical perspective, the absolute magnitude of the potential is less important than its alteration in response to a given process or perturbation.

Membrane dipole potential modifiers, such as polyphenols, alkaloids, saponins, certain hormones, synthetic dyes, and a number of drugs, including anesthetics and phosphodiesterase type 5 inhibitors, are amphiphilic molecules that alter the dipole potential by disrupting the lipid packing or orientation of interfacial water. These compounds serve as valuable experimental tools for probing the role of dipole potential in ion channel kinetics and protein function. Among them, certain biomolecules are of particular interest due to their low toxicity. Our understanding of such modifiers and their mechanisms of action continues to grow, underscoring the need for periodic review and systematic documentation of these molecular tools for potential biotechnological and pharmacological applications. Moreover, a detailed understanding of structure–activity relationships, particularly how specific chemical moieties influence the dipole potential, enables a more targeted approach to discovering and designing new modifiers.

## Data Availability

No new data were created or analyzed in this study. Data sharing is not applicable to this article.
